# Globe-LFMC, a global plant water status database for vegetation ecophysiology and wildfire applications

**DOI:** 10.1038/s41597-019-0164-9

**Published:** 2019-08-21

**Authors:** Marta Yebra, Gianluca Scortechini, Abdulbaset Badi, María Eugenia Beget, Matthias M. Boer, Ross Bradstock, Emilio Chuvieco, F. Mark Danson, Philip Dennison, Victor Resco de Dios, Carlos M. Di Bella, Greg Forsyth, Philip Frost, Mariano Garcia, Abdelaziz Hamdi, Binbin He, Matt Jolly, Tineke Kraaij, M. Pilar Martín, Florent Mouillot, Glenn Newnham, Rachael H. Nolan, Grazia Pellizzaro, Yi Qi, Xingwen Quan, David Riaño, Dar Roberts, Momadou Sow, Susan Ustin

**Affiliations:** 1 Fenner School of Environment & Society, Australian National University, Canberra, ACT Australia; 2 Bushfire & Natural Hazards Cooperative Research Centre, Melbourne, Victoria Australia; 3 School of Environment and Life Sciences, University of Salford, Salford, UK; 4 Instituto de Clima y Agua, INTA. Hurlingham, Buenos Aires, Argentina; 5 Hawkesbury Institute for the Environment, Western Sydney University, Sydney, NSW Australia; 6 University of Wollongong, Wollongong, NSW Australia; 7 Department of Geology, Geography and the Environment, University of Alcala, Alcala de Henares, Madrid Spain; 8 Department of Geography, University of Utah, Salt Lake City, USA; 9 Universitat de Lleida, Lleida, Spain; 10 CSIR, NRE, Stellenbosch, South Africa; 11 CSIR Meraka Institute, Pretoria, South Africa; 12 Laboratoire des Ressources Sylvo-Pastorales, Institut Sylvo Pastoral de Tabarka, 8110 Jendouba, Tunisia; 13 School of Resources and Environment, University of Electronic Science and Technology of China, Sichuan, China; 14 Rocky Mountain Research Station, Fire Sciences Laboratory, USFS, Montana, USA; 15 Nelson Mandela University, School of Natural Resource Management, George, South Africa; 16 Environmental Remote Sensing and Spectroscopy Laboratory (SpecLab), Spanish National Research Council (CSIC), Madrid, Spain; 17 UMR CEFE, CNRS, université de Montpellier, Université Paul Valery Montpellier, EPHE, IRD, 1919 route de mende, 34293 Montpellier Cedex 5, France; 18 CSIRO, Clayton South, Victoria Australia; 19 Istituto di Biometeorologia (Sassari) Consiglio Nazionale delle Ricerche (CNR-IBIMET), Sassari, Italy; 20 University of Nebraska-Lincoln, Lincoln, Nebraska USA; 21 Center for Spatial Technologies and Remote Sensing, UC-Davis, Davis, USA; 22 Department of Geography, University of California, Santa Barbara, USA; 23 Institut des Sciences de l’Environnement (ISE), Faculté des Sciences et Techniques, Université Cheikh Anta Diop de Dakar, Dakar, Senegal

**Keywords:** Forest ecology, Natural hazards

## Abstract

Globe-LFMC is an extensive global database of live fuel moisture content (LFMC) measured from 1,383 sampling sites in 11 countries: Argentina, Australia, China, France, Italy, Senegal, Spain, South Africa, Tunisia, United Kingdom and the United States of America. The database contains 161,717 individual records based on *in situ* destructive samples used to measure LFMC, representing the amount of water in plant leaves per unit of dry matter. The primary goal of the database is to calibrate and validate remote sensing algorithms used to predict LFMC. However, this database is also relevant for the calibration and validation of dynamic global vegetation models, eco-physiological models of plant water stress as well as understanding the physiological drivers of spatiotemporal variation in LFMC at local, regional and global scales. Globe-LFMC should be useful for studying LFMC trends in response to environmental change and LFMC influence on wildfire occurrence, wildfire behavior, and overall vegetation health.

## Abstract (summary)

**Table:** 

Design Type(s)	database creation objective • cross validation objective • physiological process monitoring objective
Measurement Type(s)	moisture content trait
Technology Type(s)	digital curation
Factor Type(s)	geographic location • environmental feature
Sample Characteristic(s)	Earth (Planet) • United States of America • French Republic

Machine-accessible metadata file describing the reported data (ISA-Tab format)

## Background & Summary

Live Fuel Moisture Content (LFMC) is the water content of live foliage relative to its dry mass, which influences vegetation susceptibility to wildfire^1^. Vegetation with high LFMC takes longer to ignite and leaf water acts as a heat sink, slowing down the rate of fire spread and reducing fire intensity^2,3^. LFMC as a measure of plant water status also has important implications for assessing drought stress in natural vegetation^4^, determining over and under watering practices in agricultural crops^5^, and assessing vegetation health^6^ and wildlife habitat suitability^7^.

Field sampling and gravimetric methods are the most direct way to estimate LFMC. These methods require *in situ* destructive collection of a representative sample of leaf/shoot material, which is then weighed fresh, oven-dried and reweighed to determine dry matter mass. Field sampling is labour-intensive and sampling sites must be carefully selected to represent spatial variation in LFMC and vegetation types. Sampling must also be repeated over time to capture temporal variation in LFMC. Consequently, the compilation of a database capturing broad-scale spatial and temporal variability in LFMC is not feasible with the resources of a single organization or research group. Remote sensing data provide the opportunity to predict LFMC over large areas at fine spatial and temporal resolutions, but these data also require field samples for calibration and validation^1^. Given the large cost of collecting field measurements of LFMC over large areas or long time periods, an international effort to compile and share existing field observations in a global database would help overcome a key constraint for the improvement and validation of LFMC remote sensing methods.

Individual universities, research centres and government departments have started to organize and share their time series of field-sampled LFMC data. For example, the U.S. National Fuel Moisture Database^8^ (NFMD, http://www.wfas.net/nfmd/public/index.php) is a web-based query system that enables users to view live- and dead- fuel moisture data. Chuvieco^9^ made available a database (FMC_UAH v1.1, http://www.geogra.uah.es/emilio/FMC_UAH.html) composed of 880 LFMC samples taken at different campaigns from 1996 to 2010 in Spain. Since 1996, the French National Forest Service (ONF) has been sampling and freely sharing weekly LFMC (www.reseauhydrique.dpfm.fr) on 35 geolocalized sites, recently quality-checked and made available by Duché *et al*.^10,11^. However, while a large number of LFMC datasets have been published in the refereed literature, much of the source data has not previously been made available to the research community.

**Fig. 1:**
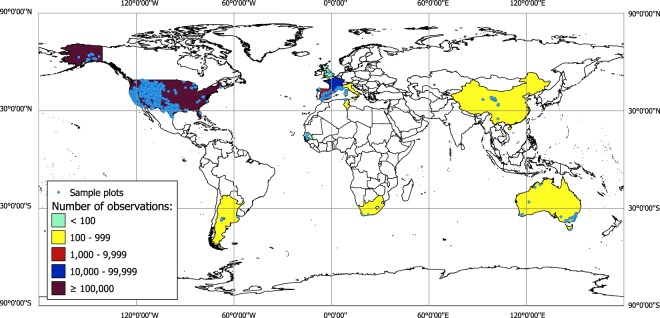
Geographical distribution of LFMC samples in Globe-LFMC. Sample plots locations, and the number of observation included in the database per country as indicated by colours in the legend (Figure created with QGIS^19^). A majority of samples were collected in the Western US, France, Spain and Australia.

We present Globe-LFMC^12^, the most comprehensive global database of *in situ* destructive sampling measurements of LFMC. Globe-LFMC is a compilation of 161,717 field measurements carried out at 1,383 sampling sites in 11 countries from 1977 to 2018 (Fig. 1). The database is properly documented, georeferenced and publicly accessible. When available, each record has an accompanying reference. We have made all names of sampling sites and species names consistent. We have also removed duplicates and corrected inconsistencies in the LFMC data. Additionally, the database reports on the protocol used to obtain each LFMC value. Finally, we also used remote sensing to assess the heterogeneity of vegetation greenness surrounding site coordinates, since highly heterogeneous areas within a specific satellite footprint may not be suitable for the calibration or validation of remote sensing products).

This database will lead to further advances in modeling and monitoring of spatial and temporal variation in LFMC. It should also allow evaluation of LFMC estimation methods, providing guidance for end-users in determining which LFMC estimation methods best fit their specific application. The database will also assist investigation of spatial variability in LFMC across the plant, local, and regional scales, and allow improved sampling strategies to capture spatial and temporal variation. The database can also be used to calibrate dynamic global vegetation models, eco-physiological models of plant drying as well as understanding the environmental and physiological drivers of LFMC. Finally, the database may be useful for exploring LFMC trends in response to environmental change and LFMC influence on wildfire occurrence, wildfire behaviour and overall vegetation health.

## Methods

Globe-LFMC unifies existing LFMC data created and provided by researchers and agencies in different countries (Fig. 1). All of the data presented in the database were collected by *in situ* destructive sampling of leaf material or, occasionally, small twigs (<0.6 cm). After the mass of fresh samples was determined, samples were dried in an oven until the water was evaporated, and then the sample was reweighed to determine dry mass. LFMC is typically calculated as the percentage of water mass with respect to dry mass, and can thus be over 100%.

The sampling methods for the different data sources were slightly different in terms of the equipment used to collect the samples, the drying temperature and time and other protocols for data acquisition or processing. Globe-LFMC summarizes the sampling methods used via a unique code (see Data Records paragraph), and the detailed description of the methodology can be found in the citation of each code included in the database.

Overall, only the LFMC from leaves or small terminal twigs (<0.6 cm) was considered and added to the database. Occasionally information about other vegetation components was recorded in the source data but this information was omitted in Globe-LFMC because leaves are generally the dominant component when viewing vegetation from above and thus contribute the most to the spectral signal observed by an airborne/spaceborne sensor. Moreover, any quality control flags in the source data indicating low-quality data led to the omission of the corresponding LFMC values in Globe-LFMC.

LFMC values from samples corresponding to the same date, species and site were recorded as a mean value. This approach allowed us to maintain consistent information on every single species sampled at the same plot. However, in some instances, the sampler collected and weighed different species together in the same sample. In those situations, the “Species collected” database field contains a list of species instead of a single species. Sometimes the species were reported with their common names or with typos in the original datasets, in which case the correct genus and species was substituted.

There were some entries where the same site name was used for more than one set of geographic coordinates. In order to have a single LFMC value for each species-date-site combination, the names were modified by adding an identifier (e.g. an increasing number or the state abbreviation). Conversely, we found some entries where two or more different site names corresponded to identical geographic. Those names were unified creating a new name (e.g. from “A” and “B” to “A–B”, where A and B are two different names, or from “C1” and “C2” to “C”, where C corresponds to the word that was in common in the C1 and C2 names). Plots with missing geographic coordinates were not added to the final database.

Most of the information provided came from the original datasets, but a few columns were added to the database to provide additional insight into site characteristics. A column was added for “Land Cover” that provides information on the land cover class at the sample site obtained from the 2015 ESA Climate Change global land cover map at 300 m spatial resolution for the year 2015 (http://maps.elie.ucl.ac.be/CCI/viewer/download.php). Columns were also added for “NDVI SD_min_”, “NDVI SD_max_”, “NDVI CV_min_” and “NDVI CV_max_”. These refer to the minimum and maximum Standard Deviation and the Coefficient of Variation of the Normalised Difference of Vegetation Index (NDVI)^13^ within a 500 m square buffer centred on the geographic coordinate of each site. These NDVI-derived statistics were computed as indicators of the heterogeneity of the sampling sites and the area surrounding them. Filtering out heterogeneous sites may be a key site selection criteria for calibration and validation of LFMC predictions from coarser spatial resolution remote sensing products. Both NDVI Standard Deviation and Coefficient of Variation were computed from Landsat 8 Operational Land Imager data using Google Earth Engine^14^. Monthly mean NDVI maps were created using *USGS Landsat 8 Surface Reflectance Tier 1* data and masking the pixels marked as cloud, cloud shadows, or snow, for each month for 2015 (the same year of the ESA land cover map used for the characterization of the land cover type of each site). For each of the 12 monthly maps, the standard deviation and the mean were computed within the 500 m × 500 m window. If 20% or more of NDVI values within the window were missing due to cloud and snow masking, no NDVI value was reported for that month. Consequently, every site was assigned 12 NDVI standard deviation values (one for each month) and 12 NDVI mean values. Globe-LFMC contains the minimum and maximum Standard Deviation and the Coefficient of Variation of NDVI values of every site. Finally, we also added information on slope and altitude for some sampling plots.

### Description of google earth engine code

Google Earth Engine^14^ was used to compute the NDVI statistics added to Globe-LFMC. The input of the program is a point shapefile (“samplePlotsShapefile”, extensions.cpg, .dbf, .prj, .shp, .shx) representing the location of each Globe-LFMC site. This shapefile is available as additional data in figshare^12^ (see Code Availability). To run this GEE code the shapefile needs to be uploaded into the GEE Assets and, then, imported into the Code Editor with the name “plots” (without quotation marks).

The outputs of the program are 12 “.csv” files, each corresponding to a month of the year 2015. Every file contains the following statistics for the 500 × 500 m^2^ buffers around the coordinates of the Globe-LFMC site: NDVI SD, NDVI mean, the count of total pixels and the unmasked pixels.

The computation of NDVI statistics is performed on U.S. Geological Survey Landsat 8 Surface Reflectance Tier 1 images (https://developers.google.com/earth-engine/datasets/catalog/LANDSAT_LC08_C01_T1_SR).

## Data Records

**Fig. 2:**
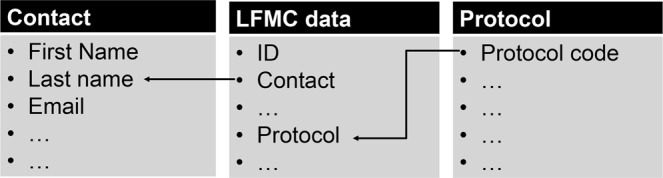
The linkage between Globe-LFMC spreadsheets.

The compiled data are available in a single database in Excel format with three different interrelated spreadsheets; “Contact”, “LFMCdata” and “Protocol^12^” (Fig. 2). A description of the fields in each spreadsheet can be found in Tables 1, 2 and 3. Each data record represents the LFMC measurement taken at a sampling site (Sitename) at a specific time and has a unique record identification code: C(contact_id)_(Sitename _id)_(record_id). These details allow users to select and download discrete datasets for their area of interest, and to identify the contact person for each data entry.

**Table 1:** Column description of the Contact Details spreadsheet.

Field	Description
First name	First name of the contact person.
Last name	Last name of the contact person.
Email	E-mail address of the person to be contacted.
Tel (include all codes)	Phone number of the person to be contacted.
Institution	The institution where the contact person works.
Address	Address of the institution.
Country	Country of the institution.
Web page	Link to the institutional profile page (if available) of the person to be contacted.

**Table 2:** Column description of the LFMC data spreadsheet.

Field	Description
ID	Unique sample ID: C(contact)_(sitename)_(record)
Contact	Last name of the person to be contacted for this dataset.
Sitename	Descriptive title of the sampling area.
State/Region	State or region where the sampling area is located.
Country	Country where the sampling area is located.
Latitude	Latitude of sampling area location (Decimal Degrees, DD).
Longitude	Longitude of sampling area location (Decimal Degrees, DD).
Sampling time	Time when the sampling occurred (hh: mm, 24-hour notation)
Sampling date	Date when the sampling occurred, in the format “yyyymmdd”.
Sampling year	Year when the sampling occurred.
Protocol	Identifier of the protocol used to obtain the LFMC value. Details present in the “LFMC protocol codes” spreadsheet.
Land Cover	Land cover type of the sampling area in the year 2015, according to ESA Climate Change Initiative – Land Cover (UCLouvain 2017).
LFMC value	Live Fuel Moisture Content value.
Units	Unit of measurement of LFMC. 1 for %, 2 for g/m^2^, 3 for others.
NDVI SD min	Minimum Standard Deviation of monthly averaged Landsat 8 OLI NDVI in the year 2015, computed on an area of 500 m × 500 m around the site’s coordinates.
NDVI SD max	Maximum Standard Deviation of monthly averaged Landsat 8 OLI NDVI in the year 2015, computed on an area of 500 m × 500 m around the site’s coordinates.
NDVI CV min	Minimum Coefficient of Variation (CV) of monthly averaged NDVI in the year 2015, computed on an area of 500 m × 500 m around the site’s coordinates.
NDVI CV max	Maximum Coefficient of Variation (CV) of monthly averaged NDVI in the year 2015, computed on an area of 500 m × 500 m around the site’s coordinates.
Species collected	Scientific name of the species sampled. In the case of multiple species collected altogether: list of scientific names of the main species sampled.
Elevation (m.a.s.l)	Elevation in metres of the sampling plot.
Slope (%)	Percentage slope of the sampling plot.
Reference	Citation or link to original LFMC data sets or to relevant publications that use the data or describe the site (in cases where the collection of data in an area proceeded after the publication cited, the record in Globe-LFMC was still linked to the article related to the older sampling campaign, in order to provide a description of the site).
Name of picture file	Name of the photograph showing the sampling site.

**Table 3:** Column description of the protocol codes file.

Field	Description
Protocol code	ID corresponding to the one contained in the “LFMC data” sheet
Time range for sampling	Time range of sampling.
New and old leaves combined	Whether the Fuel Moisture Content value is a combination of current and previous year leaves.
Weighing procedure	1 if the fresh samples were weighed on the field; 2 if the fresh samples were weighed in the lab.
Weighing device accuracy (g)	Accuracy (g) of the weighing device used.
Material for transportation	Material or equipment used to seal the samples brought to the laboratory.
Drying device	1 if oven; 2 if microwave
Drying time (h)	Duration (in hours) of the drying procedure of the sample.
Drying temperature (°C)	Temperature in Celsius degrees of the drying procedure.
Observations	Further comments and information regarding the protocol used.

We plan to publish updates to LFMC-Globe as new data become available in the future. Scientists interested in sharing their data can contact the corresponding author of this manuscript to get instructions on how to share their data.

## Technical Validation

The database represents a range of countries and land cover types containing LFMC values that range from 0.21–549% (Table 4). A majority of samples were collected in the Western US, due to extensive government sampling programs for assessing wildfire danger, with some time series stretching back decades. Large numbers of samples were also collected in France, Spain and Australia (Table 4). The land cover type with the largest number of observations and sites is “Tree cover, needle-leaved, evergreen, closed to open (>15%)” followed by “Shrubland”, “Grassland” and “Cropland-rainfed” (Table 5).

**Table 4:** Distribution of dataset records and descriptive statistics for LFMC by country and overall (Global).

Country	Sites	*N*	Years range	LFMC Min	LFMC Max	LFMC Mean	LFMC Median	Dominant Land Cover (number of observations)	Dominant Land Cover (number of plots)
Global	1,383	161,717	1977–2018	0.21	549.21	106.07	98.00	Tree cover, needleleaved, evergreen, closed to open (>15%)	Tree cover, needleleaved, evergreen, closed to open (>15%)
Argentina	17	183	2008–2010	6.94	272.22	90.68	78.70	Shrubland	Shrubland
Australia	42	673	2005–2016	5.90	473.00	109.39	105.75	Tree cover, broadleaved, evergreen, closed to open (>15%)	Tree cover, broadleaved, evergreen, closed to open (>15%)
China	229	229	2013–2016	52.37	323.44	174.53	172.05	Grassland	Grassland
France	36	20,099	1996–2018	9.99	215.90	81.05	77.53	Tree cover, needleleaved, evergreen, closed to open (>15%)	Tree cover, needleleaved, evergreen, closed to open (>15%)
Italy	1	535	2005–2011	30.17	236.97	100.75	97.80	Shrubland	Shrubland
Republic of South Africa	2	138	2016–2018	64.32	181.65	114.46	108.62	Tree cover, broadleaved, evergreen, closed to open (>15%)	Shrubland & Tree cover, broadleaved, evergreen, closed to open (>15%)
Senegal	3	96	2010	14.25	348.08	134.00	108.06	Cropland, rainfed	Cropland, rainfed
Spain	76	3,608	1996–2017	0.21	549.21	94.70	82.02	Shrubland	Mosaic tree and shrub (>50%)/herbaceous cover (<50%)
Tunisia	8	358	2010–2012	39.16	220.01	107.90	98.08	Tree cover, broadleaved, evergreen, closed to open (>15%)	Tree cover, broadleaved, evergreen, closed to open (>15%)
UK	6	26	2008–2017	64.00	185.04	116.81	117.03	Shrub or herbaceous cover, flooded, fresh/saline/brakish water	Shrub or herbaceous cover, flooded, fresh/saline/brakish water
USA	963	135,772	1977–2018	1.00	490.00	109.95	100.00	Tree cover, needleleaved, evergreen, closed to open (>15%)	Tree cover, needleleaved, evergreen, closed to open (>15%)

*n* = number of observations. “Dominant Land Cover (number of observations)” and “Dominant Land Cover (number of plots)“ summarize the land cover type with more number of observations and sites, respectively, overall (Global) and per country.

**Table 5:** Distribution of dataset records and descriptive statistics by land cover type.

Land Cover Type	*N*	Plots	LFMC Min	LFMC Max	LFMC Mean	LFMC Median
Tree cover, needleleaved, evergreen, closed to open (>15%)	78554	546	1.00	477.00	106.81	100.00
Shrubland	51833	312	2.00	467.00	104.19	93.52
Grassland	9074	280	0.52	549.21	111.20	103.00
Cropland, rainfed	6068	25	6.70	456.00	88.85	79.55
Mosaic tree and shrub (>50%)/herbaceous cover (<50%)	3240	31	2.66	350.33	86.11	77.44
Tree cover, mixed leaf type (broadleaved and needleleaved)	3070	28	14.00	468.00	119.18	108.00
Tree cover, broadleaved, deciduous, closed to open (>15%)	2848	27	0.70	357.53	110.03	100.00
Tree cover, needleleaved, evergreen, closed (>40%)	1704	39	12.00	416.00	130.94	119.00
Mosaic cropland (>50%)/natural vegetation (tree, shrub, herbaceous cover) (<50%)	1678	14	0.21	490.00	112.47	92.00
Tree cover, broadleaved, evergreen, closed to open (>15%)	701	26	39.73	307.19	119.33	111.20
Tree cover, flooded, fresh or brakish water	684	6	8.00	335.00	130.97	132.00
Tree cover, needleleaved, deciduous, closed to open (>15%)	563	4	21.00	404.00	119.38	105.00
Mosaic natural vegetation (tree, shrub, herbaceous cover) (>50%)/cropland (<50%)	523	15	7.00	280.00	106.54	102.00
Herbaceous cover	299	9	10.39	520.85	136.98	102.00
Mosaic herbaceous cover (>50%)/tree and shrub (<50%)	272	1	76.00	300.00	141.25	131.00
Urban areas	244	2	39.00	255.00	121.67	118.00
Water bodies	169	1	58.00	286.00	104.21	97.00
Sparse vegetation (tree, shrub, herbaceous cover) (<15%)	88	2	70.00	278.41	129.29	120.00
Bare areas	51	1	44.81	220.01	110.51	92.57
Shrub or herbaceous cover, flooded, fresh/saline/brakish water	26	6	64.00	185.04	116.81	117.03
Shrubland deciduous	24	5	20.27	129.00	50.73	41.19
Tree or shrub cover	2	1	88.67	116.83	102.75	102.75
Unconsolidated bare areas	2	2	104.19	131.50	117.85	117.85

n = number of observations.

**Fig. 3:**
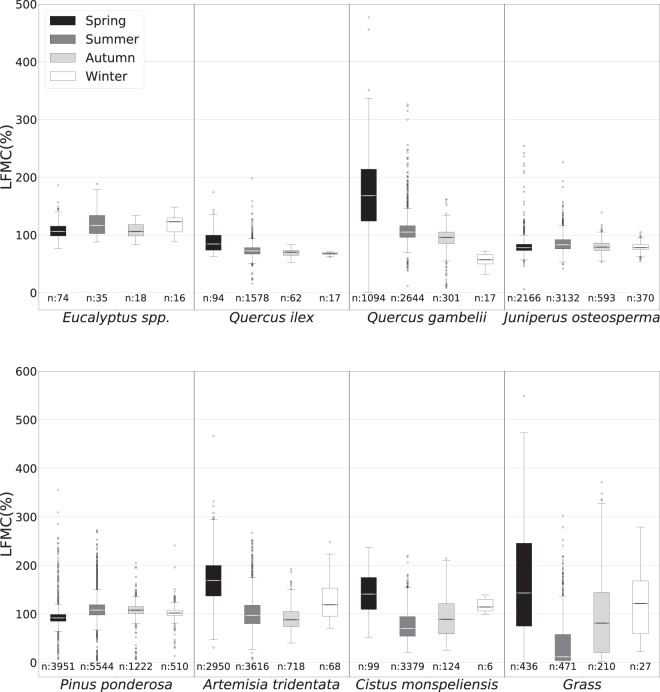
Boxplots representing the seasonal LFMC of some of the species with more observations. The number of observations used to compute each box has been added. The range of dates used to identify each season was defined using the astronomical Universal Time-based equinoxes and solstices. For the countries of the northern hemisphere, Spring was considered to be between 20 March and 19 June, Summer between 20 June and 21 September, Autumn between 22 September and 20 December, Winter between 21 December and 19 March; while, in the southern hemisphere, Spring was considered to be between 22 September and 20 December, Summer between 21 December and 19 March, Autumn between 20 March and 19 June, Winter between 20 June and 21 September. (Boxplots created with Matplotlib^20^ library).

Data in the database have been checked for possible replications and errors. We validated the data by checking their consistency with expected LFMC ranges, noting that it is out of the scope of this paper to provide detail LFMC trend analysis as this will be the objective of future work. Globe-LFMC contains values lower than 30% which are specific to dead fuels^15^. Those values mostly come from partially or fully cured grassland and herbaceous plots but were also occasionally recorded in other landcovers (Table 4 and Fig. 3). If we don’t take into account those occasional outliers, the distribution of LFMC for species with significant numbers of observations (Fig. 3) shows consistency with established knowledge on the seasonal pattern of LFMC according to the type of vegetation, their strategies to cope with drought^16^ and their pyro-ecophysiological traits^17,18^. For example, *Eucalyptus* is a genus which includes over seven hundred species of broad-leaved trees, usually evergreen and native to Australia. Because *Eucalyptus* trees have roots up to more than 2.5 m in length and adapted ecophysiological traits, they can draw water from deep in the soil profile to avoid drought and therefore their LFMC only fluctuates around a value of 100% across seasons. Similarly, *Quercus ilex* is an evergreen broad-leaved oak native of the Mediterranean region with similar strategies. Conversely, *Quercus gambelii* is a deciduous broad-leaved tree widespread in western North America that shows greater LFMC variability, with values in summer significantly lower than in spring. Finally, *Artemisia tridentata* (drought deciduous/evergreen shrub of western North America), *Cistus monspeliensis* (evergreen Mediterranean shrub) and grasslands present the strongest seasonality with the highest values in spring, lowest (<30%) in summer and intermediate in autumn and winter.

## Usage Notes

Users of the database are encouraged to look at the available photos of the sites, whose names can be found in the “LFMC data” spreadsheet. The photos are contained in the zip folder named “photos of sites”.

If the database is to be used for remote sensing products calibration or validation, fields for minimum and maximum “NDVI SD” and “NDVI CV” are recommended to be explored for a selection of the most homogenous sites.

An extra database in Excel format (“References&Changes_LFMC.xlsx”, at figshare)^12^ with two spreadsheets “References” and “Changes to USA National FM db” contain information on references, copyright notices and list the changes to the original datasets. We provide this information in case a researcher would like to compare the values shown here to the original databases.

## ISA-Tab metadata file

Download metadata file

## Data Availability

The Google Earth Engine (GEE)^14^ code and the shapefile “samplePlotsShapefile” (extensions.cpg, .dbf, .prj, .shp, .shx), used for computing the NDVI statistics are part of the data and files uploaded together with Globe-LFMC into figshare^12^. This code can only be run if the user has access to a Google account and to GEE.
