# Author Correction: Globe-LFMC, a global plant water status database for vegetation ecophysiology and wildfire applications

**DOI:** 10.1038/s41597-019-0315-z

**Published:** 2019-11-29

**Authors:** Marta Yebra, Gianluca Scortechini, Abdulbaset Badi, María Eugenia Beget, Matthias M. Boer, Ross Bradstock, Emilio Chuvieco, F. Mark Danson, Philip Dennison, Victor Resco de Dios, Carlos M. Di Bella, Greg Forsyth, Philip Frost, Mariano Garcia, Abdelaziz Hamdi, Binbin He, Matt Jolly, Tineke Kraaij, M. Pilar Martín, Florent Mouillot, Glenn Newnham, Rachael H. Nolan, Grazia Pellizzaro, Yi Qi, Xingwen Quan, David Riaño, Dar Roberts, Momadou Sow, Susan Ustin

**Affiliations:** 1 Fenner School of Environment & Society, Australian National University, Canberra, ACT Australia; 2 Bushfre & Natural Hazards Cooperative Research Centre, Melbourne, Victoria Australia; 3 School of Environment and Life Sciences, University of Salford, Salford, UK; 4 Instituto de Clima y Agua, INTA. Hurlingham, Buenos Aires, Argentina; 5 Hawkesbury Institute for the Environment, Western Sydney University, Sydney, NSW Australia; 6 University of Wollongong, Wollongong, NSW Australia; 7 Department of Geology, Geography and the Environment, University of Alcala, Alcala de Henares, Madrid Spain; 8 Department of Geography, University of Utah, Salt Lake City, USA; 9 Universitat de Lleida, Lleida, Spain; 10 CSIR, NRE, Stellenbosch, South Africa; 11 CSIR Meraka Institute, Pretoria, South Africa; 12 Laboratoire des Ressources Sylvo-Pastorales, Institut Sylvo Pastoral de Tabarka, 8110 Jendouba, Tunisia; 13 School of Resources and Environment, University of Electronic Science and Technology of China, Sichuan, China; 14 Rocky Mountain Research Station, Fire Sciences Laboratory, USFS, Montana, USA; 15 Nelson Mandela University, School of Natural Resource Management, George, South Africa; 16 Environmental Remote Sensing and Spectroscopy Laboratory (SpecLab), Spanish National Research Council (CSIC), Madrid, Spain; 17 UMR CEFE, CNRS, université de Montpellier, Université Paul Valery Montpellier, EPHE, IRD, 1919 route de mende, 34293 Montpellier, Cedex 5 France; 18 CSIRO, Clayton South, Victoria, Australia; 19 Istituto di Biometeorologia (Sassari) Consiglio Nazionale delle Ricerche (CNR-IBIMET), Sassari, Italy; 20 University of Nebraska-Lincoln, Lincoln, Nebraska USA; 21 Center for Spatial Technologies and Remote Sensing, UC-Davis, Davis, USA; 22 Department of Geography, University of California, Santa Barbara, USA; 23 Institut des Sciences de l’Environnement (ISE), Faculté des Sciences et Techniques, Université Cheikh Anta Diop de Dakar, Dakar, Senegal

Correction to: *Scientific Data* 10.1038/s41597-019-0164-9, published online 21 August 2019

Following publication of this Data Descriptor, it was found that the method for thresholding inclusion of monthly Normalised Difference of Vegetation Index (NDVI) values in the Globe-LFMC dataset was incorrectly stated as >80% of values in a given window missing due to cloud and snow masking. This has now been corrected in the HTML and PDF versions.

In addition, due to a datum assignment error, values included in Table 5 were incorrectly reported. Table 5 has been corrected in the HTML and PDF versions, and the underlying data corrected in the associated figshare record.

